# Supplementary material for: Productivity costs associated with reactive school closures related to influenza or influenza-like illness in the United States from 2011 to 2019
Source: PLoS One. 2023 Jun 6;18(6):e0286734. doi: 10.1371/journal.pone.0286734 (PMC10243616; doi:10.1371/journal.pone.0286734)
Supplement: S1 File — (DOCX) [file pone.0286734.s001.docx]

**S1. Equations for estimating productivity costs of parents, teachers, and non-teaching school staff**

$$Productivity Cost of Parents=\left[ \left( number of students\div average number of children per household \right) \right] \times fraction of households with children whose parents missed work \times average hourly wages of parents by state and year\times8 hours\times\left( 1+ nonwage benefits rate \right)\times number of days of unplanned school closure$$

$$Productivity Cost of Teachers =number of teachers \times average daily wages of teachers by school grade, state, and year \times\left( 1+nonwage benefits rate \right)\times number of days of school closure$$

$$Productivity Cost of Non-teaching School Staff =number of students\div ratio of students to school staff by state and year \times average daily wages of school staff by school grade, state, and year \times\left( 1+nonwage benefits rate \right)\times number of days of school closure$$
