# Supplementary material for: Productivity costs associated with reactive school closures related to influenza or influenza-like illness in the United States from 2011 to 2019
Source: PLoS One. 2023 Jun 6;18(6):e0286734. doi: 10.1371/journal.pone.0286734 (PMC10243616; doi:10.1371/journal.pone.0286734)
Supplement: S1 Table — (DOCX) [file pone.0286734.s002.docx]

**S2. Parameters values and assumptions for productivity costs estimation**

| **Parameter Description** | **Base Value** | **Range** | **Source** |
| --- | --- | --- | --- |
| State- and year-specific average hourly wages for parents  (2019 USD; BLS code 00-0000) | - 2012: $18.9‒$40.7 - 2013: $19.0‒$40.6 - 2014: $19.2‒$40.8 - 2015: $19.5‒$41.6 - 2016: $19.6‒$42.5 - 2017: $19.5‒$43.0 - 2018: $19.3‒$43.0 - 2019: $19.3‒$43.2   (varies by state) | NA | [1] |
| State- and year-specific average annual wages for elementary and middle school teachers (2019 USD; BLS code 25-2021) | - 2012: $44,429‒$82,744 - 2013: $44,335‒$80,154 - 2014: $44,287‒$80,809 - 2015: $45,042‒$81,650 - 2016: $43,833‒$82,372 - 2017: $42,273‒$84,003 - 2018: $41,182‒$84,512 - 2019: $44,060‒$82,830   (varies by state) | NA | [1] |
| State- and year-specific average annual wages for high school teachers (2019 USD; BLS code 25-2031) | - 2012: $44,618‒$82,544 - 2013: $44,478‒$82,579 - 2014: $45,853‒$82,807 - 2015: $46,337‒$86,881 - 2016: $45,228‒$87,368 - 2017: $43,681‒$89,093 - 2018: $43,310‒$86,844 - 2019: $44,610‒$87,240   (varies by state) | NA | [1] |
| Weighted average of national daily wage for non-teaching school staff (2019 USD, varies by year) | $56,646‒$59,495 | NA | [1-3] |
| Staff-student ratio | 1:8 | NA | [4] |
| Average children per household | 1.75 | NA | [5] |
| Fraction of households with children who missed work during unplanned school closures | 20% [6] | 10%–45% | [6-11] |
| Nonwage benefits as a fraction of total wage + nonwage pay for employees (varies by year) | 44.5%‒46.4% | NA | [12] |

USD: US dollars, BLS: U.S. Bureau of Labor Statistics, NA: Not applicable

**References**

1. Occupational Employment and Wage Statistics. Washington, DC: U.S. Bureau of Labor Statistics; 2022 [cited 2022 March 15]. Available from: <https://www.bls.gov/oes/current/oessrcst.htm>.

2. Digest of Education Statistics 2021. Table 213.20. Staff employed in public elementary and secondary school systems, by type of assignment and state or jurisdiction: Fall 2019. Washington, DC: National Center for Education Statistics, Institute of Education Sciences, U.S. Department of Education; 2022 [cited 2022 May 3]. Available from: <https://nces.ed.gov/programs/digest/d21/tables/dt21_213.20.asp>.

3. Digest of Education Statistics 2014. Table 213.20. Staff employed in public elementary and secondary school systems, by type of assignment and state or jurisdiction: Fall 2012 Washington, DC: National Center for Education Statistics, Institute of Education Sciences, U.S. Department of Education 2014 [cited 2022 May 6]. Available from: <https://nces.ed.gov/programs/digest/d14/tables/dt14_213.20.asp>.

4. Digest of Education Statistics 2021. Table 213.50. Staff, enrollment, and pupil/staff ratios in public elementary and secondary school systems, by state or jurisdiction: Selected years, fall 2000 through fall 2019 Washington, DC: National Center for Education Statistics, Institute of Education Sciences, U.S. Department of Education 2022 [cited 2022 May 6]. Available from: <https://nces.ed.gov/programs/digest/d21/tables/dt21_213.50.asp?current=yes>.

5. Germann TC, Gao H, Gambhir M, Plummer A, Biggerstaff M, Reed C, et al. School dismissal as a pandemic influenza response: When, where and for how long? Epidemics. 2019;28:100348. Epub 20190612. doi: 10.1016/j.epidem.2019.100348. PubMed PMID: 31235334; PubMed Central PMCID: PMCPMC6956848.

6. Steelfisher GK, Blendon RJ, Bekheit MM, Liddon N, Kahn E, Schieber R, et al. Parental attitudes and experiences during school dismissals related to 2009 influenza A (H1N1) --- United States, 2009. MMWR Morb Mortal Wkly Rep. 2010;59(35):1131-4. PubMed PMID: 20829746.

7. Timperio C, Hunbaugh K, Riggs M, Barrios L, Waller A, Deniston M, et al. Impact of seasonal influenza-related school closures on families - Southeastern Kentucky, February 2008. MMWR Morb Mortal Wkly Rep. 2009;58(50):1405-9. PubMed PMID: 20032924.

8. Russell ES, Zheteyeva Y, Gao H, Shi J, Rainey JJ, Thoroughman D, et al. Reactive School Closure During Increased Influenza-Like Illness (ILI) Activity in Western Kentucky, 2013: A Field Evaluation of Effect on ILI Incidence and Economic and Social Consequences for Families. Open Forum Infect Dis. 2016;3(3):ofw113. Epub 20160525. doi: 10.1093/ofid/ofw113. PubMed PMID: 27800520; PubMed Central PMCID: PMCPMC5084722.

9. Mizumoto K, Yamamoto T, Nishiura H. Contact behaviour of children and parental employment behaviour during school closures against the pandemic influenza A (H1N1-2009) in Japan. J Int Med Res. 2013;41(3):716-24. Epub 20130423. doi: 10.1177/0300060513478061. PubMed PMID: 23613502.

10. Johnson AJ, Moore ZS, Edelson PJ, Kinnane L, Davies M, Shay DK, et al. Household responses to school closure resulting from outbreak of influenza B, North Carolina. Emerg Infect Dis. 2008;14(7):1024-30. doi: 10.3201/eid1407.080096. PubMed PMID: 18598620; PubMed Central PMCID: PMCPMC2600319.

11. Epson EE, Zheteyeva YA, Rainey JJ, Gao H, Shi J, Uzicanin A, et al. Evaluation of an unplanned school closure in a Colorado school district: implications for pandemic influenza preparedness. Disaster Med Public Health Prep. 2015;9(1):4-8. doi: 10.1017/dmp.2015.3. PubMed PMID: 25739043.

12. Employer Costs for Employee Compensation. Washington, DC: U.S. Bureau of Labor Statistics; 2022 [updated June 17; cited 2022 March 31]. Available from: <https://www.bls.gov/bls/news-release/ecec.htm#2011>.
