# Supplementary material for: Productivity costs associated with reactive school closures related to influenza or influenza-like illness in the United States from 2011 to 2019
Source: PLoS One. 2023 Jun 6;18(6):e0286734. doi: 10.1371/journal.pone.0286734 (PMC10243616; doi:10.1371/journal.pone.0286734)
Supplement: S2 Table. Mean annual number of ILI-related reactive school closures and mean annual productivity costs, by study period — (DOCX) [file pone.0286734.s003.docx]

**S3 Table. Mean annual number of ILI-related reactive school closures and mean annual productivity costs, by study period**

|  | **2011/12‒2015/16** | **2016/17‒2018/19** | **2011/12‒2018/19** |
| --- | --- | --- | --- |
| **Mean annual number of school closure (n)** | 165 | 1,711 | 745 |
| **Mean annual productivity cost (2019 USD)** |  |  |  |
| Overall | 9,061,300 | 143,713,921 | 59,556,033 |
| School staff | 3,267,293 | 52,551,742 | 21,748,961 |
| Teachers | 2,907,257 | 44,788,458 | 18,612,707 |
| Parents | 2,886,751 | 46,373,721 | 19,194,364 |

ILI, influenza or influenza-like illness
