# Supplementary material for: Productivity costs associated with reactive school closures related to influenza or influenza-like illness in the United States from 2011 to 2019
Source: PLoS One. 2023 Jun 6;18(6):e0286734. doi: 10.1371/journal.pone.0286734 (PMC10243616; doi:10.1371/journal.pone.0286734)
Supplement: S3 Table — a Among 448 schools, 274 schools (61%) were located in Tennessee, 83 schools (19%) were in Kentucky, 19 schools (4%) in Texas, 18 schools (4%) in Michigan, and 13 schools (3%) were in Alabama. b Among 15 schools, 10 schools (67%) were located in Kentucky, 5 schools (33%) were in Tennessee. c Among 689 schools, 381 schools (55%) were located in Tennessee, 119 schools (17%) were in Kentucky, 38 schools (6%) in Texas, 37 schools (5%) in Michigan, and 30 schools (4%) were in Oklahoma. d Among 357 schools, 202 schools (57%) were located in Tennessee and 114 schools (32%) were in Kentucky. e Among 94 schools, 44 schools (47%) were located in Kentucky and 43 schools (46%) were in Tennessee. f All schools (100%) are located in Tennessee. (DOCX) [file pone.0286734.s004.docx]

**S4 Table. Number of schools with multiple ILI-related reactive school closures, from 2011-2012 to 2018-2019**

|  | n | % |  |
| --- | --- | --- | --- |
| Number of school closures in a given school year | | | |
| 1 | 3,038 | 86.8 |  |
| 2 | 448^a^ | 12.8 |  |
| 3 | 15^b^ | 0.4 |  |
| Total | 3,501 | 100 |  |
| Number of school closures from 2011-12 to 2018-19 | | | |
| 1 | 2,184 | 62.4 |  |
| 2 | 689^c^ | 19.7 |  |
| 3 | 357^d^ | 10.2 |  |
| 4 | 94^e^ | 2.7 |  |
| 5 | 155^f^ | 4.4 |  |
| 7 | 3^f^ | 0.1 |  |
| 8 | 17^f^ | 0.5 |  |
| 9 | 2^f^ | 0.1 |  |
| Total | 3,501 | 100 |  |

^a^ Among 448 schools, 274 schools (61%) were located in Tennessee, 83 schools (19%) were in Kentucky, 19 schools (4%) in Texas, 18 schools (4%) in Michigan, and 13 schools (3%) were in Alabama.

^b^ Among 15 schools, 10 schools (67%) were located in Kentucky, 5 schools (33%) were in Tennessee.

^c^ Among 689 schools, 381 schools (55%) were located in Tennessee, 119 schools (17%) were in Kentucky, 38 schools (6%) in Texas, 37 schools (5%) in Michigan, and 30 schools (4%) were in Oklahoma.

^d^ Among 357 schools, 202 schools (57%) were located in Tennessee and 114 schools (32%) were in Kentucky.

^e^ Among 94 schools, 44 schools (47%) were located in Kentucky and 43 schools (46%) were in Tennessee.

^f^ All schools (100%) are located in Tennessee.

ILI, influenza or influenza-like illness
