# Supplementary material for: Productivity costs associated with reactive school closures related to influenza or influenza-like illness in the United States from 2011 to 2019
Source: PLoS One. 2023 Jun 6;18(6):e0286734. doi: 10.1371/journal.pone.0286734 (PMC10243616; doi:10.1371/journal.pone.0286734)
Supplement: S4 Table — a Closures with missing information on the length of closures were imputed to 1-day (n = 631, 11% of total closures) based on our understanding that schools may be less likely to have reopening announcements for 1-day closures versus longer closures. b Among a total of 430 closures with ≥4 days, 195 (45%) occurred in Tennessee, 156 (36%) in Kentucky, 16 (4%) in Alabama, 12 (3%) in Missouri, and 11 (3%) in Texas. c During the eight academic years from 2011–12 to 2018–19, 5,959 ILI-related closures occurred among 3,501 schools. (DOCX) [file pone.0286734.s005.docx]

**S5 Table. Number of ILI-related reactive school closures by length of closures and academic year**

| Academic Year |  | Length of school closures (per closure) ^a^ | | | | | | | |
| --- | --- | --- | --- | --- | --- | --- | --- | --- | --- |
|  | n or % | 1-day | 2-day | 3-day | 4-day | 5-day | 6-day | 7-day | Total |
| 2011-2012 | n | 81 | 23 | 0 | 0 | 0 | 0 | 0 | 104 |
|  | % | 77.9 | 22.1 | 0.0 | 0.0 | 0.0 | 0.0 | 0.0 | 100 |
| 2012-2013 | n | 218 | 104 | 33 | 20 | 0 | 0 | 0 | 375 |
|  | % | 58.1 | 27.7 | 8.8 | 5.3 | 0.0 | 0.0 | 0.0 | 100 |
| 2013-2014 | n | 11 | 0 | 0 | 0 | 0 | 0 | 0 | 11 |
|  | % | 100.0 | 0.0 | 0.0 | 0.0 | 0.0 | 0.0 | 0.0 | 100 |
| 2014-2015 | n | 157 | 102 | 36 | 0 | 0 | 0 | 0 | 295 |
|  | % | 53.2 | 34.6 | 12.2 | 0.0 | 0.0 | 0.0 | 0.0 | 100 |
| 2015-2016 | n | 24 | 12 | 4 | 0 | 0 | 0 | 0 | 40 |
|  | % | 60.0 | 30.0 | 10.0 | 0.0 | 0.0 | 0.0 | 0.0 | 100 |
| 2016-2017 | n | 516 | 462 | 155 | 129 | 17 | 9 | 0 | 1,288 |
|  | % | 40.1 | 35.9 | 12.0 | 10.0 | 1.3 | 0.7 | 0.0 | 100 |
| 2017-2018 | n | 810 | 883 | 162 | 47 | 29 | 27 | 2 | 1,960 |
|  | % | 41.3 | 45.1 | 8.3 | 2.4 | 1.5 | 1.4 | 0.1 | 100 |
| 2018-2019 | n | 594 | 848 | 294 | 93 | 35 | 22 | 0 | 1,886 |
|  | % | 31.5 | 45.0 | 15.6 | 4.9 | 1.9 | 1.2 | 0.0 | 100 |
| Total | n | 2,411 | 2,434 | 684 | 289^b^ | 81^b^ | 58^b^ | 2^b^ | 5,959^c^ |
|  | % | 40.5 | 40.9 | 11.5 | 4.9 | 1.4 | 1.0 | 0.0 | 100 |

^a^ Closures with missing information on the length of closures were imputed to 1-day (n=631, 11% of total closures) based on our understanding that schools may be less likely to have reopening announcements for 1-day closures versus longer closures.

^b^ Among a total of 430 closures with ≥4 days, 195 (45%) occurred in Tennessee, 156 (36%) in Kentucky, 16 (4%) in Alabama, 12 (3%) in Missouri, and 11 (3%) in Texas.

^c^ During the eight academic years from 2011-12 to 2018-19, 5,959 ILI-related closures occurred among 3,501 schools.

ILI, influenza or influenza-like illness
