# Supplementary material for: Productivity costs associated with reactive school closures related to influenza or influenza-like illness in the United States from 2011 to 2019
Source: PLoS One. 2023 Jun 6;18(6):e0286734. doi: 10.1371/journal.pone.0286734 (PMC10243616; doi:10.1371/journal.pone.0286734)
Supplement: S5 Table — Row% refers to proportion of closures with urbanicity characteristics using row-specific total as denominator, and Col% refers to proportion of closures using column-specific total as denominator. * During the eight academic years from 2011–12 to 2018–19, 5,724 ILI-related closures (96% of total closures) occurred among 3,289 public schools. (DOCX) [file pone.0286734.s006.docx]

**S6 Table. Number of ILI-related reactive school closures among public schools from 2011-2012 to 2018-2019, by urbanicity of school location and length of closures**

|  | City | | Suburban | | Town | | Rural | | Total | |
| --- | --- | --- | --- | --- | --- | --- | --- | --- | --- | --- |
|  | n | Row% | n | Row% | n | Row% | n | Row% | n | Row% |
| Total | 766 | 13.4 | 592 | 10.3 | 1,278 | 22.3 | 3,088 | 53.9 | 5,724* | 100.0 |
| Closures lasting ≥4 days | 60 | 14.6 | 48 | 11.7 | 65 | 15.8 | 239 | 58.0 | 412 | 100.0 |
| By Length of closures (per closure) | | | | | | | | | | |
|  | n | Col% | n | Col% | n | Col% | n | Col% | n | Col% |
| 1-day | 422 | 55.1 | 225 | 38.0 | 429 | 33.6 | 1,214 | 39.3 | 2,290 | 40.0 |
| 2-day | 269 | 35.1 | 282 | 47.6 | 572 | 44.8 | 1,234 | 40.0 | 2,357 | 41.2 |
| 3-day | 15 | 2.0 | 37 | 6.3 | 212 | 16.6 | 401 | 13.0 | 665 | 11.6 |
| 4-day | 51 | 6.7 | 48 | 8.1 | 28 | 2.2 | 147 | 4.8 | 274 | 4.8 |
| 5-day | 9 | 1.2 | 0 | 0.0 | 17 | 1.3 | 53 | 1.7 | 79 | 1.4 |
| 6-day | 0 | 0.0 | 0 | 0.0 | 20 | 1.6 | 37 | 1.2 | 57 | 1.0 |
| 7-day | 0 | 0.0 | 0 | 0.0 | 0 | 0.0 | 2 | 0.1 | 2 | 0.0 |
| Total | 766 | 100.0 | 592 | 100.0 | 1,278 | 100.0 | 3,088 | 100.0 | 5,724* | 100.0 |

Row% refers to proportion of closures with urbanicity characteristics using row-specific total as denominator, and Col% refers to proportion of closures using column-specific total as denominator.

* During the eight academic years from 2011-12 to 2018-19, 5,724 ILI-related closures (96% of total closures) occurred among 3,289 public schools.

ILI, influenza or influenza-like illness
