# Supplementary material for: Productivity costs associated with reactive school closures related to influenza or influenza-like illness in the United States from 2011 to 2019
Source: PLoS One. 2023 Jun 6;18(6):e0286734. doi: 10.1371/journal.pone.0286734 (PMC10243616; doi:10.1371/journal.pone.0286734)
Supplement: S6 Table — * Percentages were calculated based on the sum of the row (3,289 and 456 closures, respectively). All other percentages were calculated based on the sum of each column. (DOCX) [file pone.0286734.s007.docx]

**S7 Table. Number of public schools with multiple ILI-related reactive school closures, by urbanicity of school location**

|  | City | | Suburban | | Town | | Rural | | Total | |
| --- | --- | --- | --- | --- | --- | --- | --- | --- | --- | --- |
|  | n | % | n | % | n | % | n | % | n | % |
| Total | 417 | 12.7* | 284 | 8.6* | 770 | 23.4* | 1,818 | 55.3* | 3,289 | 100.0* |
| By number of closures for each school in a given school year | | | | | | | | | | |
| 1 | 357 | 85.6 | 224 | 78.9 | 673 | 87.4 | 1,579 | 86.9 | 2,833 | 86.1 |
| 2 | 60 | 14.4 | 60 | 21.1 | 88 | 11.4 | 233 | 12.8 | 441 | 13.4 |
| 3 | 0 | 0.0 | 0 | 0.0 | 9 | 1.2 | 6 | 0.3 | 15 | 0.5 |
| ≥2 | 60 | 13.2* | 60 | 13.2* | 97 | 21.3* | 239 | 52.4* | 456 | 100.0* |
| By number of closures for each school from 2011-2012 to 2018-2019 | | | | | | | | | | |
| 1 | 263 | 63.1 | 133 | 46.8 | 466 | 60.5 | 1,127 | 62.0 | 1,989 | 60.5 |
| 2 | 55 | 13.2 | 67 | 23.6 | 196 | 25.5 | 359 | 19.7 | 677 | 20.6 |
| 3 | 51 | 12.2 | 43 | 15.1 | 60 | 7.8 | 199 | 10.9 | 353 | 10.7 |
| 4 | 0 | 0.0 | 12 | 4.2 | 23 | 3.0 | 58 | 3.2 | 93 | 2.8 |
| 5 | 48 | 11.5 | 29 | 10.2 | 23 | 3.0 | 55 | 3.0 | 155 | 4.7 |
| 7 | 0 | 0.0 | 0 | 0.0 | 0 | 0.0 | 3 | 0.2 | 3 | 0.1 |
| 8 | 0 | 0.0 | 0 | 0.0 | 2 | 0.3 | 15 | 0.8 | 17 | 0.5 |
| 9 | 0 | 0.0 | 0 | 0.0 | 0 | 0.0 | 2 | 0.1 | 2 | 0.1 |

* Percentages were calculated based on the sum of the row (3,289 and 456 closures, respectively). All other percentages were calculated based on the sum of each column.

ILI, influenza or influenza-like illness
