# Supplementary material for: Productivity costs associated with reactive school closures related to influenza or influenza-like illness in the United States from 2011 to 2019
Source: PLoS One. 2023 Jun 6;18(6):e0286734. doi: 10.1371/journal.pone.0286734 (PMC10243616; doi:10.1371/journal.pone.0286734)
Supplement: S7 Table — (DOCX) [file pone.0286734.s008.docx]

**S8 Table. Number of ILI-related reactive school closures and productivity costs from 2011-2012 to 2018-2019**

|  | Number of school closures | Productivity Costs (2019 USD) | | | |
| --- | --- | --- | --- | --- | --- |
| School year |  | Parents | Teachers | School staff | Total |
| 2011-2012 | 104 | 1,347,761 | 1,378,393 | 1,534,538 | 4,260,692 |
| 2012-2013 | 375 | 6,449,499 | 6,563,862 | 7,785,237 | 20,798,599 |
| 2013-2014 | 11 | 86,766 | 92,944 | 83,936 | 263,646 |
| 2014-2015 | 295 | 5,875,487 | 5,834,845 | 6,110,971 | 17,821,303 |
| 2015-2016 | 40 | 674,239 | 666,241 | 821,781 | 2,162,261 |
| 2016-2017 | 1,288 | 37,579,105 | 36,375,387 | 42,449,055 | 116,403,546 |
| 2017-2018 | 1,960 | 48,778,766 | 47,544,630 | 53,385,564 | 149,708,960 |
| 2018-2019 | 1,886 | 52,763,292 | 50,445,356 | 61,820,608 | 165,029,256 |
| Total | 5,959 | 153,554,916 | 148,901,658 | 173,991,690 | 476,448,263 |

ILI, influenza or influenza-like illness
