# Supplementary material for: Productivity costs associated with reactive school closures related to influenza or influenza-like illness in the United States from 2011 to 2019
Source: PLoS One. 2023 Jun 6;18(6):e0286734. doi: 10.1371/journal.pone.0286734 (PMC10243616; doi:10.1371/journal.pone.0286734)
Supplement: S8 Table — * Total productivity costs include productivity costs of parents, teachers, and non-teaching school staff. (DOCX) [file pone.0286734.s009.docx]

**S9 Table. Sensitivity analysis of productivity costs of parents and total productivity costs associated with ILI-related reactive school closures by varying the fraction of parents missing work (range: 10% to 45%)**

|  | Productivity cost of parents (2019 USD) | | | Total productivity costs* (2019 USD) | | |
| --- | --- | --- | --- | --- | --- | --- |
| School year | Base case (20%) | LL (10%) | UL (45%) | Base case (20%) | LL (10%) | UL (45%) |
| 2011‒2012 | 1,347,761 | 673,880 | 3,032,462 | 4,260,692 | 3,586,812 | 5,945,393 |
| 2012‒2013 | 6,449,499 | 3,224,750 | 14,511,373 | 20,798,599 | 17,573,849 | 28,860,473 |
| 2013‒2014 | 86,766 | 43,383 | 195,224 | 263,646 | 220,263 | 372,104 |
| 2014‒2015 | 5,875,487 | 2,937,744 | 13,219,847 | 17,821,303 | 14,883,559 | 25,165,662 |
| 2015‒2016 | 674,239 | 337,120 | 1,517,038 | 2,162,261 | 1,825,141 | 3,005,059 |
| 2016‒2017 | 37,579,105 | 18,789,553 | 84,552,986 | 116,403,546 | 97,613,994 | 163,377,428 |
| 2017‒2018 | 48,778,766 | 24,389,383 | 109,752,223 | 149,708,960 | 125,319,577 | 210,682,417 |
| 2018‒2019 | 52,763,292 | 26,381,646 | 118,717,407 | 165,029,256 | 138,647,609 | 230,983,371 |
| Total | 153,554,916 | 76,777,458 | 345,498,560 | 476,448,263 | 399,670,805 | 668,391,907 |

* Total productivity costs include productivity costs of parents, teachers, and non-teaching school staff.

ILI, influenza or influenza-like illness; LL, lower-level; UL, upper-level
