# Supplementary material for: Productivity costs associated with reactive school closures related to influenza or influenza-like illness in the United States from 2011 to 2019
Source: PLoS One. 2023 Jun 6;18(6):e0286734. doi: 10.1371/journal.pone.0286734 (PMC10243616; doi:10.1371/journal.pone.0286734)
Supplement: S9 Table — (DOCX) [file pone.0286734.s010.docx]

**S10 Table. Mean number of ILI-related reactive school closures per year and the mean total productivity cost per year, by state and by study period**

|  | **Average number of closures per year (n)** | | | **Average total productivity cost per year (2019 USD)** | | |
| --- | --- | --- | --- | --- | --- | --- |
| **State** | **2011/12‒2015/16** | **2016/17‒2018/19** | **2011/12‒2018/19** | **2011/12‒2015/16** | **2016/17‒2018/19** | **2011/12‒2018/19** |
| TN | 51.0 | 820.0 | 339.4 | 2,947,167 | 82,609,824 | 32,820,664 |
| KY | 52.0 | 282.7 | 138.5 | 3,468,643 | 27,944,236 | 12,646,990 |
| TX | 0.6 | 123.3 | 46.6 | 25,358 | 6,502,554 | 2,454,307 |
| MI | 11.2 | 95.7 | 42.9 | 419,307 | 5,075,112 | 2,165,234 |
| OK | 15.0 | 77.7 | 38.5 | 462,395 | 3,324,601 | 1,535,722 |
| AL | 0.2 | 47.3 | 17.9 | 26,155 | 3,685,264 | 1,398,321 |
| AR | 2.8 | 50.0 | 20.5 | 161,684 | 2,850,305 | 1,169,917 |
| GA | 3.8 | 11.7 | 6.8 | 478,402 | 1,761,701 | 959,639 |
| MO | 7.2 | 38.0 | 2.6 | 261,630 | 1,805,814 | 840,699 |
| ID | 1.8 | 32.3 | 13.3 | 35,110 | 1,548,790 | 602,740 |
| OH | 4.2 | 14.3 | 8.0 | 165,256 | 1,063,443 | 502,076 |
| VA | 0.2 | 15.7 | 6.0 | 1,435 | 1,112,024 | 417,906 |
| LA | 0.2 | 17.0 | 6.5 | 7,348 | 733,123 | 279,514 |
| NC | 3.2 | 12.7 | 6.8 | 90,152 | 510,638 | 247,834 |
| IL | 0.8 | 7.7 | 3.4 | 62,002 | 395,937 | 187,227 |
| MS | 0.4 | 6.3 | 2.6 | 36,370 | 321,805 | 143,408 |
| KS | NR | 11.3 | 4.3 | 0 | 348,436 | 130,664 |
| NJ | 0.8 | 2.0 | 1.3 | 55,570 | 246,436 | 127,145 |
| WI | 0.6 | 7.0 | 3.0 | 8,767 | 278,577 | 109,946 |
| WV | 2.2 | 3.0 | 2.5 | 97,232 | 127,939 | 108,747 |
| CO | 1.0 | 4.3 | 2.3 | 12,838 | 226,404 | 92,925 |
| MN | 1.2 | 2.0 | 1.5 | 38,187 | 141,137 | 76,793 |
| IN | 0.2 | 3.0 | 1.3 | 11,338 | 148,329 | 62,710 |
| NM | NR | 6.7 | 2.5 | 0 | 166,287 | 62,358 |
| NE | 0.6 | 3.7 | 1.8 | 24,522 | 102,175 | 53,642 |
| SC | 0.2 | 2.0 | 0.9 | 14,386 | 97,568 | 45,579 |
| NY | 0.2 | 2.3 | 1.0 | 3,832 | 112,110 | 44,436 |
| FL | 0.4 | 1.7 | 0.9 | 14,516 | 72,301 | 36,185 |
| WA | 1.0 | NR | 0.6 | 56,705 | 0 | 35,441 |
| MD | NR | 1.0 | 0.4 | 0 | 84,814 | 31,805 |
| PA | 0.2 | 0.7 | 0.4 | 4,738 | 70,533 | 29,411 |
| IA | 0.2 | 1.7 | 0.8 | 10,369 | 58,798 | 28,529 |
| OR | 0.2 | 0.7 | 0.4 | 20,078 | 42,588 | 28,520 |
| VT | NR | 1.3 | 0.5 | 0 | 52,341 | 19,628 |
| MA | 0.4 | NR | 0.3 | 28,374 | 0 | 17,734 |
| SD | NR | 3.0 | 1.1 | 0 | 34,879 | 13,080 |
| CA | 0.2 | 0.3 | 0.3 | 2,136 | 22,824 | 9,894 |
| NH | NR | 0.3 | 0.1 | 0 | 13,139 | 4,927 |
| ME | NR | 0.3 | 0.1 | 0 | 8,459 | 3,172 |
| CT | NR | 0.3 | 0.1 | 0 | 7,142 | 2,678 |
| AZ | NR | 0.3 | 0.1 | 0 | 5,534 | 2,075 |
| MT | 0.6 | NR | 0.4 | 3,150 | 0 | 1,969 |
| AK | NR | NR | NR | 0 | 0 | 0 |
| DC | NR | NR | NR | 0 | 0 | 0 |
| DE | NR | NR | NR | 0 | 0 | 0 |
| HI | NR | NR | NR | 0 | 0 | 0 |
| ND | NR | NR | NR | 0 | 0 | 0 |
| NV | NR | NR | NR | 0 | 0 | 0 |
| RI | NR | NR | NR | 0 | 0 | 0 |
| UT | NR | NR | NR | 0 | 0 | 0 |
| WY | NR | NR | NR | 0 | 0 | 0 |

ILI, influenza or influenza-like illness; NR, not reported
