# Supplementary material for: Productivity costs associated with reactive school closures related to influenza or influenza-like illness in the United States from 2011 to 2019
Source: PLoS One. 2023 Jun 6;18(6):e0286734. doi: 10.1371/journal.pone.0286734 (PMC10243616; doi:10.1371/journal.pone.0286734)
Supplement: S10 Table — (DOCX) [file pone.0286734.s011.docx]

**S11 Table. Annual total productivity cost per student among schools with ILI-related reactive closures from 2016‒2017 to 2018‒2019 (2019 USD)**

|  | **Overall** | **By urbanicity of school location** | | | |
| --- | --- | --- | --- | --- | --- |
| **Estimates** |  | **City** | **Suburban** | **Town** | **Rural** |
| Minimum | 17.3 | 24.3 | 25.9 | 22.2 | 17.3 |
| 25th percentile | 34.0 | 31.3 | 57.8 | 37.2 | 36.7 |
| Median | 66.3 | 39.4 | 85.8 | 67.5 | 66.9 |
| **Mean** | **99.8** | **98.6** | **112.7** | **99.2** | **98.1** |
| 75th percentile | 122.4 | 95.6 | 118.3 | 128.4 | 124.0 |
| Interquartile range | 88.4 | 64.4 | 60.5 | 91.2 | 87.3 |

ILI, influenza or influenza-like illness
