# Supplementary material for: Productivity costs associated with reactive school closures related to influenza or influenza-like illness in the United States from 2011 to 2019
Source: PLoS One. 2023 Jun 6;18(6):e0286734. doi: 10.1371/journal.pone.0286734 (PMC10243616; doi:10.1371/journal.pone.0286734)
Supplement: S11 Table — * Total number of students in the U.S. public schools (overall and by urbanicity category) were based on the National Center for Education Statistics (https://nces.ed.gov/pubs2018/2018052/tables/table_04.asp). (DOCX) [file pone.0286734.s012.docx]

**S12 Table. Total productivity costs associated with ILI-related reactive school closures from 2011‒2012 to 2018‒2019 among public schools and total number of students in U.S. public schools in 2015‒2016**

|  | Overall | By urbanicity of school location | | | |
| --- | --- | --- | --- | --- | --- |
|  |  | City | Suburban | Town | Rural |
| Total productivity costs among public schools with ILI-related closures, 2011-2012 to 2018-2019 (2019 USD) | | | | | |
| All public schools | 460,599,747 | 62,149,629 | 69,812,689 | 112,376,093 | 216,261,335 |
| Tennessee | 257,862,754 | 44,485,510 | 57,512,449 | 54,250,622 | 101,614,173 |
| Kentucky | 100,574,776 | 6,279,838 | 3,608,591 | 30,200,798 | 60,590,412 |
| Arkansas | 9,359,337 | 0 | 1,053,703 | 4,004,342 | 4,301,292 |
| Oklahoma | 12,191,411 | 1,661,003 | 154,842 | 2,718,244 | 7,657,322 |
| Idaho | 4,810,672 | 11,248 | 90,824 | 1,453,958 | 3,265,889 |
| Others (46 states) | 75,800,797 | 9,712,031 | 7,392,280 | 19,748,128 | 38,832,248 |
| Number of students in the U.S. public schools, 2015-2016 (n)* | | | | | |
| All U.S. public schools | 49,312,454 | 14,892,361 | 19,577,044 | 5,572,307 | 9,221,429 |
| Tennessee | 991,648 | 322,286 | 203,288 | 164,614 | 300,469 |
| Kentucky | 676,793 | 108,287 | 149,571 | 172,582 | 247,029 |
| Arkansas | 491,390 | 140,538 | 69,777 | 115,968 | 165,107 |
| Oklahoma | 692,546 | 164,133 | 153,745 | 163,441 | 211,227 |
| Idaho | 286,447 | 66,742 | 79,632 | 68,461 | 71,039 |
| Others (46 states) | 46,173,630 | 14,090,376 | 18,921,030 | 4,887,242 | 8,226,558 |

* Total number of students in the U.S. public schools (overall and by urbanicity category) were based on the National Center for Education Statistics (https://nces.ed.gov/pubs2018/2018052/tables/table_04.asp)

ILI, influenza or influenza-like illness
