# Supplementary material for: Productivity costs associated with reactive school closures related to influenza or influenza-like illness in the United States from 2011 to 2019
Source: PLoS One. 2023 Jun 6;18(6):e0286734. doi: 10.1371/journal.pone.0286734 (PMC10243616; doi:10.1371/journal.pone.0286734)
Supplement: S1 Fig — * No ILI-related reactive school closures were observed in public schools in the states of WY, WA, UT, RI, ND, NV, MD, HI, DC, DE, CT, CA, and AK from 2011–12 to 2018–19. (DOCX) [file pone.0286734.s013.docx]

**S13 Fig. Annual total productivity cost per student associated with ILI-related reactive school closures among all U.S. public schools, by state* and academic year (2019 USD)**


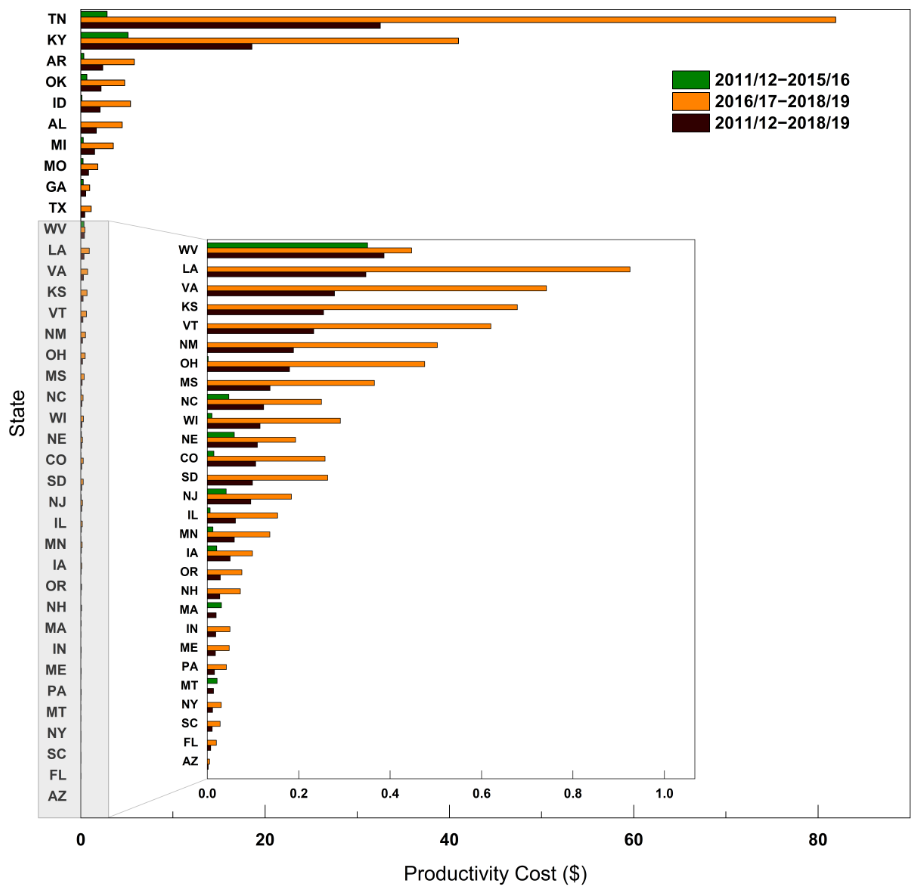


* No ILI-related reactive school closures were observed in public schools in the states of WY,WA, UT,RI, ND, NV, MD, HI, DC, DE, CT, CA, and AK from 2011‒12 to 2018‒19.

ILI, influenza or influenza-like illness
